# Access to essential cardiovascular medicines for children: a pilot study of availability, price and affordability in Nigeria

**DOI:** 10.1093/heapol/czz057

**Published:** 2019-12-09

**Authors:** Ebiowei S F Orubu, Faith O Robert, Mercy Samuel, Daniel Megbule

**Affiliations:** 1 Department of Pharmaceutics and Pharmaceutical Technology, Faculty of Pharmacy, Niger Delta University, Wilberforce Island, Bayelsa State, Nigeria; 2 Department of Biochemistry, Faculty of Basic Medical Sciences, Niger Delta University, Wilberforce Island, Bayelsa State, Nigeria

**Keywords:** Access, essential medicines, cardiovascular medicines, children, Nigeria

## Abstract

Policies to improve access to medicines for children in low- and middle-income countries, such as Nigeria, should consider the growing threat of non-communicable diseases. The aim of this pilot study was to scope availability, price and affordability of essential cardiovascular medicines for children in selected states in Nigeria. The study was a descriptive longitudinal survey conducted in three phases. Availability was determined as percentage of facilities having the medicine on the survey date. Medicines with good availability (>80%) were noted. Prices were cross-referenced against international Reference Prices and the Nigerian National Health Insurance Scheme Prices. Affordability was calculated using the Least-Paid Government Worker method. For medicines compounded to improve availability, a model for calculating affordability was proposed. In Phase I, the availability of all 17 strengths of the cardiovascular medicines or diuretics listed in the Essential Medicines List for Children (2015) were surveyed in two conveniently selected states using the WHO/HAI questionnaire. Data were collected from 17 hospitals and pharmacies. Phases II and III focused on tablet formulations (enalapril, furosemide, hydrochlorothiazide and spironolactone) in three purposively selected state capitals: Lagos, Abuja and Yenagoa. In Phase II, 11 private pharmacies were surveyed in December 2016: Phase III tracked price changes in Abuja and Yenagoa in August 2018. Only furosemide and hydrochlorothiazide tablets had good availability. Oral liquids were unavailable. Prices for four generic oral tablets were 2–16× higher than the International Reference Prices; prices for two of these did not change significantly over the study period. Affordable medicines were generic furosemide and hydrochlorothiazide tablet. Where a fee is charged, compounded medicines were also not affordable. While the small sample sizes limit generalization, this study provides indicative data suggesting that prices for cardiovascular medicines remain high and potentially unaffordable in the private sector in these selected states, and when compounded. Regular systematic access surveys are needed.

**Key Messages**

- Access to essential medicines for cardiovascular diseases in children was lower than the 80% recommendation by the World Health Organization. Age-appropriate formulations were not commercially available in the study locations. Prices of four selected oral tablet formulations were generally higher than the median International Reference Prices, ranging for 2-fold for hydrochlorothiazide to 16-fold for enalapril. Only two of these medicines—generic furosemide and hydrochlorothiazide—were affordable. However, when these had to be compounded for children younger than 5 years, where compounding pharmacies impose a compounding fee, they became unaffordable.
- Nigeria is committed to providing Universal Health Coverage. While government seeks to extend insurance coverage, it may also be necessary to guarantee access to a basic basket of medicines for people who still or have to pay for medicines out of pocket to ensure financial protection. Subsidies may be considered for paediatric medicines that need to be compounded.

## Introduction

Access to medicines, under Universal Health Coverage (UHC), is an integral target of the Sustainable Development Goals (SDGs) which propose a global developmental roadmap for 2015–30. In 2016, Nigeria committed to achieving UHC by implementing a new National Health Policy. Yet, health insurance coverage, while it has expanded recently to include both federal and state insurance schemes, is still low, with vulnerable populations having only limited financial protection. With an estimated population of 191 million, Nigeria is currently the world’s seventh most populous country and is projected to become the third by 2050 (United Nations Department of Economic and Social Affairs, Population Division, 2017). Children below the age of 14 years are the majority (44%) of Nigeria’s population. However, the development indices are poor for children, with Nigeria having one of the world’s highest burden of under-five mortality arising from a lack of access to medicines for preventable and treatable communicable diseases (UNICEF, 2014).

The burden of cardiovascular diseases in children in Nigeria, and other African countries, is thought to be higher than in other parts of the world (Zühlke *et al.*, 2013; Stewart *et al.*, 2016). Specific studies in Nigeria have estimated the overall prevalence of cardiovascular diseases in the under-five to be as high as 10–35% (Sani *et al.*, 2007; Emodi *et al.*, 2014). Congenital Heart Diseases—ventricular septal defects or patent ductus arteriosus, e.g.—with current estimated prevalence of ≈1%, is rising (Chinawa *et al.*, 2013; Otaigbe and Tabansi, 2014; Arodiwe *et al.*, 2015; Abdulkadir and Abdulkadir, 2016). Acquired heart diseases that arise as sequelae of infectious diseases such as rheumatic fever, renal disease, or the inherited sickle-cell disease—20% of children with sickle-cell disease have pulmonary hypertension—are prevalent (Wilson et al, 2014; Musa *et al.*, 2016; Animasahun *et al.*, 2018). Cardiovascular diseases, whether congenital in aetiology or acquired, resulting in conditions such as pulmonary hypertension, oedema (hydrocephalus) and heart failure are associated with high morbidity and mortality in children. Thus, Nigeria faces a double burden of disease (Emodi *et al.*, 2014).

Essential medicines are defined as those required to meet the priority healthcare needs of the population (WHO, 2016). These medicines are contained in the World Health Organization’s (WHO’s) Essential Medicines List (EML), and the Essential Medicines List for children (EMLc). Medicines for cardiovascular disease and oedema, as contained in the EMLc, are: digoxin, dopamine, enalapril, furosemide, hydrochlorothiazide, mannitol and spironolactone. These seven Active Pharmaceutical Ingredients (APIs) are listed as 12 formulations (tablets, oral and parenteral liquids) in 17 strengths. Of these, digoxin, furosemide and spironolactone have tablets as well as oral liquid formulations; dopamine and mannitol are parenterals; and enalapril and hydrochlorothiazide are listed only as tablets (WHO, 2015). The age-appropriateness of medicines require that patients of all age-groups get medicines suited to their ability and capability (EMA, 2013). Young children, 0–5 year olds, cannot safely swallow conventional tablets; and are traditionally administered oral medicines as liquid formulations (Standing and Tuleu, 2005). Currently, the WHO recommends flexible solid oral dosage forms such as dispersible tablets as the preferred formulations for children (World Health Organization, 2012). These are dosage forms that could be given either as oral liquids to young children who cannot safely swallow tablets, or as intact tablets to older children. Essential medicines should be made available in age-appropriate dosage forms to guarantee access for all patient groups, including young children.

Access to medicines is a multidimensional concept covering commercial availability, affordability, quality, geographical availability and rational use (Wirtz *et al.*, 2016). The commercial availability of age-appropriate dosage forms varies from country to country; but is more likely to be greater in developed countries where most product innovation occur (Orubu and Tuleu, 2017). The lack of age-appropriate formulations, *ceteris paribus*, makes a medicine inaccessible to all. Worldwide, access to cardiovascular medicines for children is sub-optimal, where access in this case refers to the commercial availability of age-appropriate medicines (Wimmer *et al.*, 2014). The other dimensions of access to cardiovascular medicines for children are rarely studied, especially for low- and middle-income countries, such as Nigeria (The World Bank, 2016).

While one would hypothesize the limited availability and affordability of medicines in a lower-middle-income country with very limited insurance coverage, it is still important to collect evidence on the extent of the problem to support the need for policy reform. Current data on access to medicines is not available. Availability, price and affordability surveys for a broad basket of medicines in Nigeria were last conducted in 2004. There is no specific survey on cardiovascular medicines for children.

The aim of this pilot study was to provide current indicative data on the availability, price and affordability of essential cardiovascular medicines for children in selected states in Nigeria.

## Methods

### Study design

The study was designed as a descriptive survey employing the WHO/HAI survey methodology (Health Action International, 2018). It was conducted in three phases. Phase I scoped the availability of all essential cardiovascular medicines and diuretics as listed in the EMLc, 2015; while in Phase II, prices and affordability of selected medicines was surveyed. Phase III was a follow-up study of phase II repeated 18–20 months later to track changes in price and affordability. In Phases II and III, surveyed medicines were all for oral use, and were selected based on the findings from Phase I.

### Survey instrument

The survey instrument in Phase I was the WHO/HAI survey questionnaire made up of three sections. Section I surveyed background information about the facility: location, name, type of outlet and survey date. Section II included information about the medicines surveyed: generic or brand name, strength and dosage form, pack size, and unit cost. Section III collected information about the responder providing the price and availability data.

In Phases II and III, the mystery shopper approach was employed where the researcher walked into purposively and conveniently selected pharmacies to request for the four selected oral cardiovascular medicines and to purchase those available; or to ask for their prices. The rationale for the choice of pharmacies was location to a main secondary or tertiary hospital which were known to the researcher to either handle cardiovascular conditions in children or were the foremost tertiary hospital in the state.

### Survey locations

The phase I study of availability was conducted in two conveniently selected states, out of 37 federating units (36 states and 1 Federal Capital Territory, FCT), to represent two out of six geopolitical zones in Nigeria. These states were Delta and Oyo State. In each selected state, the capital city and one other city within an hour’s journey by bus from the capital was surveyed. In each of these two states, 10 facilities (five in each city) made up of the main public tertiary hospital, where available, or public secondary hospitals or private hospitals and community pharmacies about an hour’s bus ride from the main tertiary hospital were randomly selected to be surveyed. Survey locations included both urban and rural areas. While these locations were conveniently selected, certain features must be noted about their roles in the management of childhood diseases or position in the medicines supply chain of Nigeria. Oyo State contains a foremost referral facility in Nigeria, the University College Hospital, located in the capital city, Ibadan; while the capital of Delta State, Asaba, lies only a 5-min bus ride from one of the largest open drug markets in Nigeria—the Onitsha Bridgehead market in Anambra State. By including both states, it was intended to survey both this hospital and the open drug market.

In the Phase II study of price and affordability, three different states were surveyed: Lagos State, the FCT and Bayelsa State, respectively, in the South-West, North-Central and South-West, geopolitical zones of Nigeria, representing three out of the six zones. The rationale for selection of these states were similar to the rationale for the phase I scoping survey. Lagos State contains a mix of some of the best health facilities in Nigeria as well as being home to a thriving drug market and major licensed pharmaceutical distributors; hence was an ideal location for the purposes of this study. It was also the first capital of Nigeria. The current capital, Abuja, had the first National Hospital for Women and Children in the country, hence, was also thought necessary. The assumption here was that, together, Lagos State and the FCT would represent ideal locations for the study, as places where if any medicine is obtainable in Nigeria, it must be in these. These locations were, thus, considered ‘major markets’. Bayelsa State, in contrast, is a much smaller and newer state with fewer specialized medical facilities and pharmaceutical outlets. It was, thus, chosen to represent a ‘small market’ in Nigeria. The facilities surveyed in these states were all in the capital cities, respectively, Lagos, Abuja and Yenagoa, as per WHO/HAI recommendation; noting too that the capital cities have the greater concentrations of well-equipped health facilities and well-stocked pharmacies. While Lagos and Abuja are urban areas, Yenagoa can be described as peri-urban. In the Phase III follow-up survey, only Bayelsa State and FCT were included, as a result of financial considerations. Almost all of the same pharmacies that were surveyed in the phase II study were visited. These phases were intended as some follow-up study to one conducted earlier with a focus on hospitals (Orubu *et al.*, 2017).

The sample size selected, or the number of facilities surveyed in each location, was limited to the major hospitals or pharmacies in each location based on the knowledge of the researchers.

### Medicines surveyed

In phase I, all 12 formulations of the INNs listed as essential cardiovascular medicines or diuretics: digoxin, dopamine, enalapril, furosemide, hydrochlorothiazide, mannitol and spironolactone were surveyed to scope availability. In Phases II and III a focused sub-set of four formulations for oral use: enalapril, furosemide, hydrochlorothiazide and spironolactone tablet were surveyed. This set was based on availability data from phase I which showed them to be the most common formulations.

### Availability

Availability was calculated as percentage of surveyed facilities with the medicine on the day of the survey. Based on this percentage, availability was described using the criteria of Wang *et al.* (2014) as: ‘not available’ (0%), ‘low’ (<50%), ‘fairly high’ (50–80%) or ‘good’ (>80%). Availability is described for all facilities and expressed as a composite for each state.

### Prices

Prices were expressed as Median Price Ratio (MPR) which was calculated by comparing the median generic medicine price in United States Dollars (USD), following conversion from the local currency, the Naira (N), using the historic exchange rate, with the median International Reference Prices (IRP) for the same formulations in the MSH’s International Medical Products Price Guide using data from 2015 (Abrha *et al.*, 2018; Management Sciences for Health, 2018; Khuluza and Haefele-Abah, 2019). A rate of N350 to 1USD was assumed as the historical rate for the middle of 2016, when data collection commenced. Prices were also compared against the Nigerian National Health Insurance Scheme Price List (2013).

### Affordability

The Least-Paid Government Worker (LPGW) method was adopted in which affordability is determined as the amount of days’ pay needed to procure a month’s treatment. The wage for the LPGW at the time of the study was 18 000 naira per month, or 600 naira ($1.7)/day for a 30-day month, which is the minimum wage. It was assumed that one dosage unit (one tablet/injection ampoule/infusion bag) in the dosage strengths given in the EMLc would provide a day’s dose; therefore, 30 of such units would provide a month’s supply. Formulations were categorized into brands, branded generics and generics. This method was used for calculating affordability for children aged 5 years and above who can safely swallow conventional-sized tablets.

For children under the age of 5 years who cannot safely swallow intact conventional-sized tablets, and for whom oral liquids are compounded/prepared by crushing tablets and dispersing in a suspending vehicle, affordability was calculated as shown in Table 1. A medicine is said to be affordable if the total paid for the compounding of 1 month’s supply of the medicines costs at most a day’s wage.

**Table 1:** Estimated total cost and assumptions for compounding essential cardiovascular medicines that are not commercially available in Nigeria, 2016

INN	Tablet strength in mg (a)	Most commonly compounded strength, MCCS, in mg/ml (b)^a^	Dosing frequency as number of times per day (c)^b^	Usual dose for neonates in mg/kg body weight (e)^c^	Median dose for a neonate in mg/kg body weight, obtained from e (f)	Average body weight of a neonate in kg (g)^d^	Total daily dose in mg, which is g x f x c (h)	Total monthly dose in mg is h*30 (i)	Total volume of a Month's supply of MCCS, or 30 x h/b (j)	No. of tablets required is j/a (k)	Cost of one tablet in USD (m)^e^	Total cost of tablets required, or k*m (n)	Total cost of compounding liquid required,^f^ or 0.57* j/100 (*P*)	Total for a month's supply, or (2 + *n* + *P*)^g^, in USD
Furosemide	40	1	1	0.5–2	1	3.5	3.5	105	105	3	0.2	0.6	0.6	3.2
Enalapril	5	1	1	0.01–0.5	0.25	3.5	0.875	26.25	26	5	0.1	0.5	0.1	2.6
HCT	25	1	1	1–2	1.5	3.5	5.25	157.5	158	6	0.1	0.6	0.9	3.5
Spironolactone	25	1	1	1–2	1.5	3.5	5.25	157.5	158	6	0.2	1.2	0.9	4.1

Notes and assumptions:

a For uniformity this was assumed to be 1 mg/ml for all INN.

b For uniformity, this was also assumed to be one.

c Data from the British National Formulary for children, 2015; except for hydrochlorothiazide for which there is no dosing information. For HCT, therefore, the dosing schedule for spironolactone was adopted, since they tend to be given together in the local setting (unpublished data).

d As obtained from the British National Formulary for children, 2015.

e As obtained from survey results.

f The unit cost of one 100 ml volume of compounding liquid was estimated at 200 naira, or 0.57USD, for the commonly used suspending vehicles of vitamin B complex or vitamin C syrup (Orubu *et al.*, 2017).

g The two stands for the cost of compounding, in USD; assumed to be uniform across all compounding locations.

HCT, hydrochlorothiazide.

### Data collection

Availability data were collected between June and August 2016. In Delta State, results from two facilities were incomplete, so only eight facilities were included in the data analysis. These were made up of two public secondary hospitals, two private hospitals and four community pharmacies. The open drug market could not be surveyed as there was no one willing to share price information. In Oyo State, three public hospitals made up of one tertiary and two secondary and six community pharmacies were surveyed. Thus, a total of 17 facilities were surveyed in the two states.

Price and affordability data for Phase II were collected in December 2016. Eleven facilities were included in the survey, and a total of 36 samples were obtained. In Phase III, price and affordability data were collected in August 2018 from five pharmacies: two in Yenagoa, and three in Abuja.

### Data analysis

All facilities surveyed were anonymized. Results are descriptive and expressed as percentage for availability and ratios for prices and affordability.

## Results

### Availability

**Figure 1:**
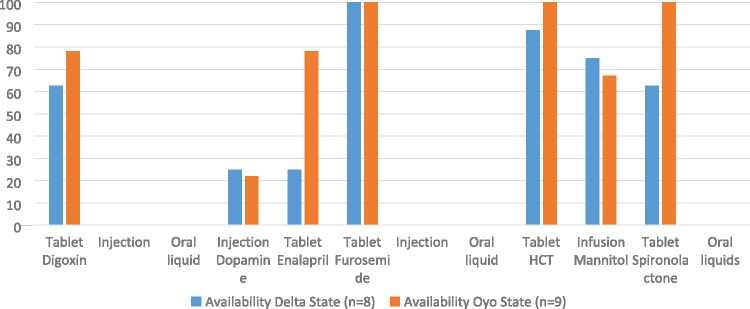
Availability of the 12 formulations of essential cardiovascular medicines and diuretics in two selected states in Nigeria, June to August 2016. Tablets were the more commonly available formulations. Furosemide and hydrochlorothiazide tablets were the only formulations with good availability (>80%). Spironolactone tablet’s availability was variable, with good availability only in Oyo State. There were no oral formulations in the sampled facilities.

In phase I, of all 12 formulations in the EMLc, 2015, only two—furosemide and hydrochlorothiazide tablets—had good availability (>80%) in the two states surveyed. Furosemide tablet was the only medicine available (100%) in all the sampled facilities. Age-appropriate oral liquid formulations of digoxin, furosemide and spironolactone were not available; as were the injection formulations of digoxin and furosemide (Figure 1). Availability was the same for 67% (10/12) formulations in the two states; with enalapril and spironolactone tablets having better availability in Oyo State compared with Delta State. For both States, dopamine injection had poor availability.

Specifically, in Delta State, the availability of individual formulations ranged from 25% to 100%. Furosemide tablet was found in all the surveyed outlets (100% availability) while dopamine and enalapril had the lowest availability (25%) each. Hydrochlorothiazide tablet had good availability (87.5%). Digoxin and spironolactone tablets had fairly high availabilities (62.5% each), as with mannitol (75%).

### Price

**Figure 2:**
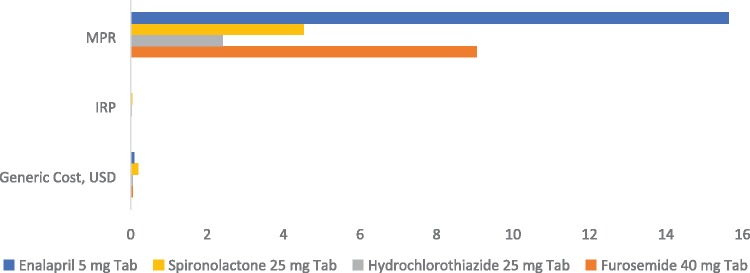
Price comparison using MPRs of four oral cardiovascular medicines from 17 health facilities (public and private hospitals and private pharmacies) across three states in Nigeria against the International Median Buyers Price, or IRP. (Note: Prices are in USD based on the Naira-US Dollar rate of June 2016; while IRP prices are as December 2015, the most current update.) The medicines were all more expensive than the IRP, with unit costs ranging from 2× IRP for hydrochlorothiazide to 16× for enalapril.

The prices of all four oral generic medicines in the phase II study were 2- to 16-folds higher than the IRP: hydrochlorothiazide (2.4-fold), spironolactone (5-fold), furosemide (9-fold) and enalapril (16-fold) (Figure 2). Originator brands were more expensive than generics; with mean originator prices across all locations higher than the generics by 1.3× for spironolactone, 4.8× for furosemide and 10.8× for hydrochlorothiazide. Enalapril was available only as the generic. Local prices were still higher when the IRP price data were broken down into generic and brands. Interestingly, unit prices (price per tablet) for generic furosemide, hydrochlorothiazide and spironolactone were found to be higher than prices in the Nigerian National Health Insurance Scheme Price List (2013), by respectively 14×, 7× and 5×. Enalapril was not included in the Scheme. At the prices included in the Scheme, all the medicines listed were affordable.

Prices for spironolactone and hydrochlorothiazide generic tablets did not change significantly in five pharmacies in two study locations, Yenagoa and Abuja, when assessed 18 months later.

### Affordability

Generic furosemide and hydrochlorothiazide tablets were the only affordable medicines (Table 2). The compounded oral liquid preparations were estimated as unaffordable, with a month’s supply ranging from $2.67 for enalapril to $4.16 for spironolactone (Table 1).

**Table 2:** Affordability of essential cardiovascular medicines and diuretics in a sample of selected states (Bayelsa, Lagos and the Federal Capital Territory) in Nigeria calculated using the Lowest-Paid Government Worker approach at the historic Naira-United States Dollar rate of June, 2016

Generic tablet formulation	Number of day’s wages in USD required to buy 1 month supply	WHO/HAI benchmark of affordability (no. of day’s wage needed for 1-month treatment)
Enalapril	1.7	1
Furosemide	1.0	1
Hydrochlorothiazide	0.5	1
Spironolactone	3.5	1

Medicines with median prices above 1-day’s wage are unaffordable.

## Discussion

This study is the first attempt to describe the availability, price and affordability of essential cardiovascular medicines for children in Nigeria. The results indicate that access to these medicines were sub-optimal in the study locations. While the WHO sets a target of 80% availability of all essential medicines, geographical availability of individual formulations of cardiovascular medicines and diuretics in the EMLc 2015 were as low as 17% in Oyo State and 25% in Delta State. Worthy of note is the fact that age-appropriate oral formulations were not available in the study locations. This finding corroborates the results from a survey of compounding in hospitals in Nigeria which found that cardiovascular medicines were mostly compounded because of a lack of age-appropriate formulations (Orubu *et al.*, 2017). The reasons for these observations are not completely understood. One possible reason may be the lack of commercial availability of age-appropriate medicines locally and/or of knowledge about availability in other countries. As earlier mentioned, Nigeria has a significantly lower proportion of registered age-appropriate oral medicines for children when compared against countries considered to be product innovators (Orubu and Tuleu, 2017). The low availability of most formulations in the study locations may also be explained by low demand, as pharmacies may not stock medicines for which there is no demand. These findings highlight the need for improved commercial availability of age-appropriate formulations.

Cardiovascular medicines for oral use were found to be more expensive than the International Reference Prices as well as recommended list prices in the Federal Health Insurance Scheme in the study locations. In this regard, these results conform to an earlier price survey in 2004 which found that medicine prices in Nigeria were 2–38 times higher than the International Reference Prices (Federal Ministry of Health, 2006). The reason for higher prices in Nigeria, as indicated in this pilot survey, may be due to the fact that these medicines, apart from generic hydrochlorothiazide tablet, were not manufactured in the country but imported from manufacturers in the UK, India or China. The devaluation of the naira in February 2015 saw an increase in exchange rates from about N200 for 1USD in January 2015 to about N280 to 1USD as at July 2015 in the parallel market, an increase of 40%, even though the official rate was held at N199 (The Cable, 2015). This placed an upward pressure on the prices of all imported goods, including medicines. In 2017, the government proposed a 20% duty on imported medicines. Combined with increases in the exchange rate (N360 to the USD, as at May 2018), this had led to much higher prices of medicines, and shortages of some imported drugs. It was thus assumed that prices would be much higher as at August 2018, when the survey was repeated. Surprisingly, there were no significant changes in prices (data not shown) for generic spironolactone and hydrochlorothiazide in the surveyed locations in Abuja and Yenagoa. For the two other medicines, the locations now had different generic products or pack sizes; thus, the price information was not relevant. However, this finding is not conclusive due to the sample size; a more representative longitudinal price survey is needed. Prices in private pharmacies are known to be higher than in public hospital pharmacies because public hospitals generally apply lower mark-ups. This may be one factor responsible for the price difference on comparison with the insurance listed prices.

Generic furosemide and hydrochlorothiazide tablets were the only affordable medicines, out of the four oral products, in the private facilities surveyed. In contrast, at the health insurance scheme listed prices, all four tablets were all affordable. Compounding, while it may improve geographical access to suitable medicines, may not necessarily make a medicine more affordable. Using the new formula/method employed for assessing affordability of medicines that need to be manipulated before administration to young children, all four compounded oral liquids were found to be unaffordable. At the assumed modal fee of $2 per compounding, a month’s supply of each medicine was already more than the daily wage of the LPGW. At least one study suggests that having oral liquid formulations of these medicines locally manufactured might not necessary make them all affordable, although this would depend on context (Probst and Welch, 2017). Thus, compounding may seem the most appropriate approach to increase access in these settings, provided quality can be assured. One way to increase access may be the removal of compounding fees and making extemporaneous preparations of these medicines free at compounding hospital pharmacies.

In the EMLc 2015, the only medicine listed for the treatment of hypertension in children is enalapril. However, it does not include an age-appropriate formulation for this INN, even though an oral formulation had been available since 2013 in the USA (Prnewswire, 2018). An age-appropriate formulation of enalapril should be considered for inclusion in the next edition of the EMLc, due for publication in 2019. This would guide local inclusion of the formulation in countries’ essential medicine lists.

There may be need to regulate prices for a basket of medicines. This small pilot study suggests that medicine prices remain high and potentially unaffordable. While government seeks to extend insurance coverage, it may also be necessary to guarantee access to a basic basket of medicines for people who still have to pay for medicines out-of-pocket to ensure financial protection. A regular and more comprehensive prices survey in much the same way as prices are monitored for inflation in the country may be necessary.

### Limitations

As this was designed as a pilot to generate rapid data, study site selection, sampling technique and sample size were based on researchers’ knowledge of the country and were not sufficiently robust. However, selection to ensure that the facilities surveyed are either referral hospitals for cardiovascular diseases in children, or the foremost tertiary health facility in the selected location, or are community pharmacies close to these hospitals, it is believed, made for a fairly representative sample for the availability of medicines for the condition studied. Additionally, the inclusion of two ‘large markets’ and one ‘small market’ was intended to provide representative information for the whole country. However, it is recognized that these results may still not be generalizable to the whole country. Further work is needed to generate more representative data.

There were some challenges in obtaining price data as the mystery shopper approach could not be used in all locations and availability data could not be obtained from one targeted location because of privacy concerns. The phase III study was also limited by the fact that some of the pharmacies, on the repeat visits, did not have the same product brand or generics, or were stock-out.

The views of pharmacists and staff at the surveyed facilities were not systematically gathered. This would have provided more detailed information on reasons for poor availability at these facilities.

Affordability calculation for oral compounded medicines may not apply in all cases as many different strengths of medicines are usually compounded. Additionally, the study assumed that medicines for cardiovascular conditions in children may be chronic, requiring continuous drug management. However, this may not be the case for all conditions. Thus, affordability calculations may have been over-estimated.

## Conclusions

The Nigerian government is committed to providing UHC. However, as this pilot study suggests, access to essential cardiovascular medicines for children was poor at the study locations. Age-appropriate oral dosage forms were not available in the surveyed facilities. Prices remain high, and oral compounded medicines were unaffordable. Subsidies for compounded medicines and price controls on a basket of children medicines including for cardiovascular conditions may be necessary to increase access to age-appropriate cardiovascular medicines for children in Nigeria. There is need for more comprehensive access to medicines surveys to inform health policies in the era of the SDGs.

*Conflict of interest statement*. None declared.
